# Gas Permeability of Cellulose Aerogels with a Designed Dual Pore Space System

**DOI:** 10.3390/molecules24152688

**Published:** 2019-07-24

**Authors:** Kathirvel Ganesan, Adam Barowski, Lorenz Ratke

**Affiliations:** Institue of Materials Research, German Aerospace Center (DLR), Linder Höhe, 51170 Köln, Germany

**Keywords:** cellulose aerogel, dual pore system, gas permeability, surfactant, macropore, mesopore

## Abstract

The gas permeability of a porous material is a key property determining the impact of the material in an application such as filter/separation techniques. In the present study, aerogels of cellulose scaffolds were designed with a dual pore space system consisting of macropores with cell walls composing of mesopores and a nanofibrillar network. The gas permeability properties of these dual porous materials were compared with classical cellulose aerogels. Emulsifying the oil droplets in the hot salt–hydrate melt with a fixed amount of cellulose was performed in the presence of surfactants. The surfactants varied in physical, chemical and structural properties and a range of hydrophilic–lipophilic balance (HLB) values, 13.5 to 18. A wide range of hierarchical dual pore space systems were produced and analysed using nitrogen adsorption–desorption analysis and scanning electron microscopy. The microstructures of the dual pore system of aerogels were quantitatively characterized using image analysis methods. The gas permeability was measured and discussed with respect to the well-known model of Carman–Kozeny for open porous materials. The gas permeability values implied that the kind of the macropore channel’s size, shape, their connectivity through the neck parts and the mesoporous structures on the cell walls are significantly controlling the flow resistance of air. Adaption of this new design route for cellulose-based aerogels can be suitable for advanced filters/membranes production and also biological or catalytic supporting materials since the emulsion template method allows the tailoring of the gas permeability while the nanopores of the cell walls can act simultaneously as absorbers.

## 1. Introduction

Aerogels of cellulose (AC) are one of the bio-based lightweight open porous materials. They have randomly interconnected nanofibrillar networks and a wide range of pore sizes composed of meso- and macropores [1,2,3]. In recent years, after modifying chemically or physically, the research development on cellulose aerogels has gained much interest in many research fields including the preparation of biocomposites [4,5], template or supporting materials [6], separation techniques [7,8,9], tissue engineering and medicine [10,11]. The chemical modification of cellulose surface functional groups is mainly developed for the strong physical and chemical interactions with the desired components, thermal stability and rot resistance [12,13,14,15]. On the other hand, the physical modifications of cellulose aerogels can produce useful structural properties which can enhance the mechanical properties and improve the permeability of gases and fluids. For instance, bringing in hierarchical dual pore structures, combining interconnected macro- and mesoporous, improves the elastic modulus of the aerogels of cellulose scaffolds in comparison with the cellulose aerogels which have the same density and porosity [16,17].

For catalysis, filtering and separation technologies and storage applications, the resistance of permeability and transport of molecules of different matter is considered as an important factor. The physical modifications of aerogels, incorporating macropore channels as an additional pathway to the classical porous network of cellulose aerogels can reduce the flow-resistance. Therefore, designing aerogels with hierarchical porous structures in different scale is significant. Hence understanding of the gas permeability at different hierarchical levels of pores can provide insight in engineering the materials for desired applications. The cellulose aerogels containing hierarchical dual pore structures can be synthesized by employing different sacrificial template methods [16,17,18,19] where frozen ice, porogen particles or surfactant-stabilized oil droplets act as structural template producing macropores replicating the shape of the templates. The modification of size and shape of the templates can yield the diversified porous structures.

In the present paper, we demonstrate the analysis of permeability of gas (atmospheric air) through the hierarchical dual porous structures of cellulose aerogels and discuss the data with respect to the well-known model of Carman–Kozeny for open porous materials. The dual porous structures were designed with emulsion oil template method and the pore channels were tuned and modified by varying the surfactants. This study provides understanding of the gas permeability of cellulose-based aerogel materials. Designing the hierarchical porous structure and understanding the mass transport through the open porous cellulose aerogel network is significantly important in engineering new types of filters, since the emulsion template method allows the tailoring of the gas permeability and the nanopores of the cell walls act as absorbers.

## 2. Results and Discussion

We used six surfactants with different hydrophilic–lipophilic balance (HLB) values in the range from 13.5 to 18 according to Griffin’s method to classify non-ionic surfactants. Molecules with an HLB value between 13 and 15 are detergents and emulsify oil in water, whereas value larger than 15 are solubilizing agents and are hydrophilic, meaning they are able to finely disperse the oil in water solutions. In the synthesis of aerogels of cellulose scaffold (ACS) materials, emulsifying the oil droplets with surfactants of varying hydrophilic–lipophilic balance (HLB) values in the molten salt hydrate with a fixed amount of cellulose (4 wt%) resulted in the formation of a hierarchical dual pore structure.

### 2.1. Physical Properties of Aerogels

The physical properties of cellulose scaffolds are summarized in Table 1 and Table 2. After supercritical drying, the cellulose concentration of aerogels, AC-2, AC-4 and AC-6 showed linear dependency with the concentration of cellulose, i.e., increasing the concentration of cellulose increased the envelope density of the aerogels. This characteristic behavior was observed in other polysaccharide aerogels. The ACS samples had envelope densities in the range 55–67 kg/m^3^ which were close to a similar value comparing with AC-2. The total porosity of aerogels of cellulose showed a decreasing trend while increasing the concentration of cellulose. In the case of the ACS samples, the total porosity values were almost same (96%) which were again close to the value of AC-2.

The BET isotherm linear plot was reported in one of our previous reports [17] which was a type IV isotherm according to IUPAC nomenclature. The specific surface area of all the aerogels was observed to be in the range between 287 and 327 m^2^/g, except scaffolds ACS-PN17 and ACS-PS18 showing lower values of 262 and 245 m^2^/g respectively. It was reasoned out as the effect of strong physical interactions of hydrophilic surfactants with cellulose chains while assembling the molecular chains into nanofibers [17].

### 2.2. Microstructure of Aerogels of Cellulose

**Figure 1:**
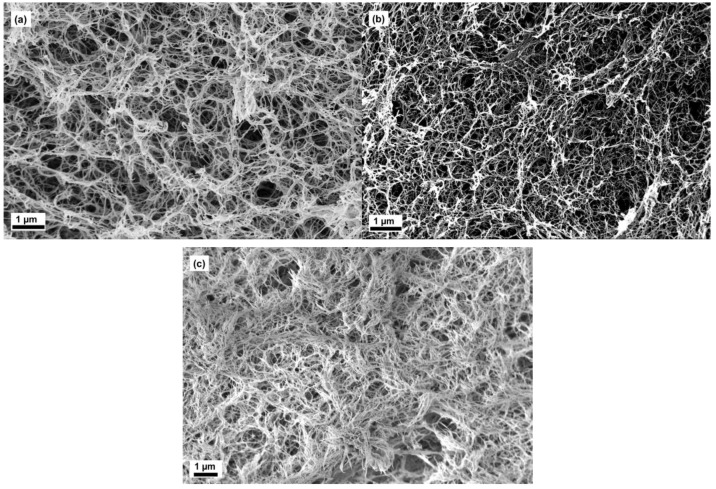
Scanning electron microscopic images of aerogels of cellulose (AC)-2 (**a**), AC-4 (**b**) and AC-6 (**c**).

The different surfactants had a pronounced effect on the microstructure. They lead to different hierarchical structures and properties as summarized in Table 2. The various microstructures were nicely visualized using scanning electron microscopy. In general all the aerogels showed open porous networks due to supercritical drying and the samples were confirmed to be pure cellulose aerogels, following the procedure reported in literature [20]. The microstructures of aerogels are shown in Figure 1.

The images of AC-2, AC-4 and AC-6 showed finely distributed nanofibrillar structures with meso- and macropores (few tens of nanometer to 1 µm). It was clearly observed that the macropore sizes were not more than 1 µm. Taking comparison of AC-2 and AC-6, the aerogels yielded the tightly packed interconnected nanofibrillar network through an increase in the concentration of cellulose.

**Figure 2:**
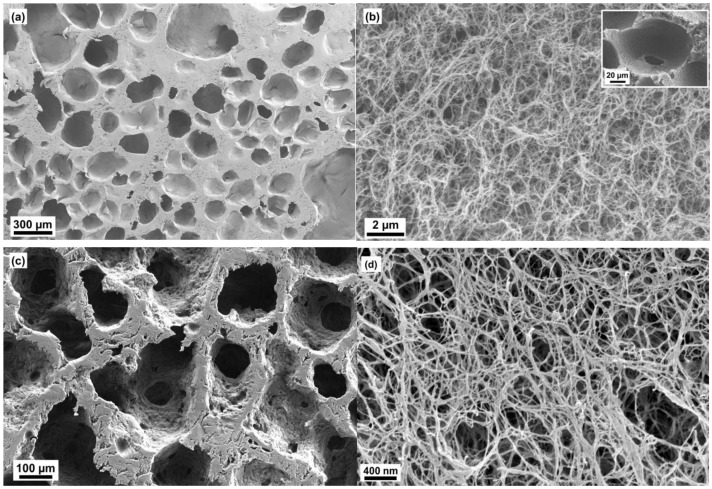
Scanning electron microscopic images of aerogels of cellulose scaffold (ACS)-PT13.5 (inset shows one of the neck part) (**a,b**) and ACS-PO15 (**c,d**).

**Figure 3:**
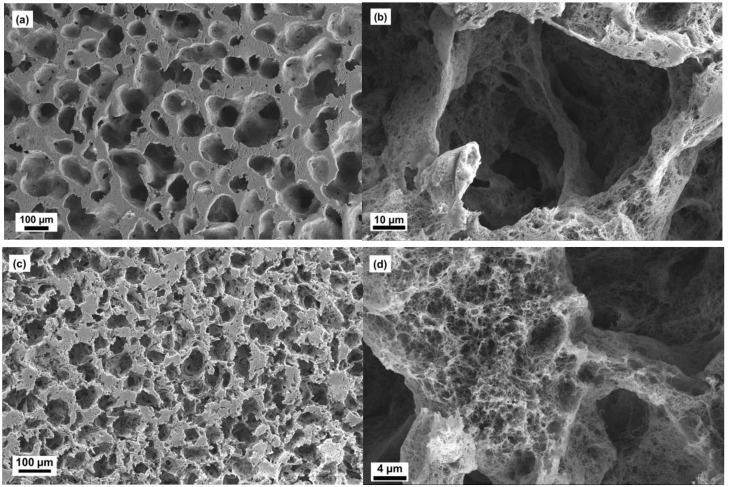
Scanning electron microscopic images of ACS-PS15 (**a,b**) and ACS-PC16 (**c,d**).

**Figure 4:**
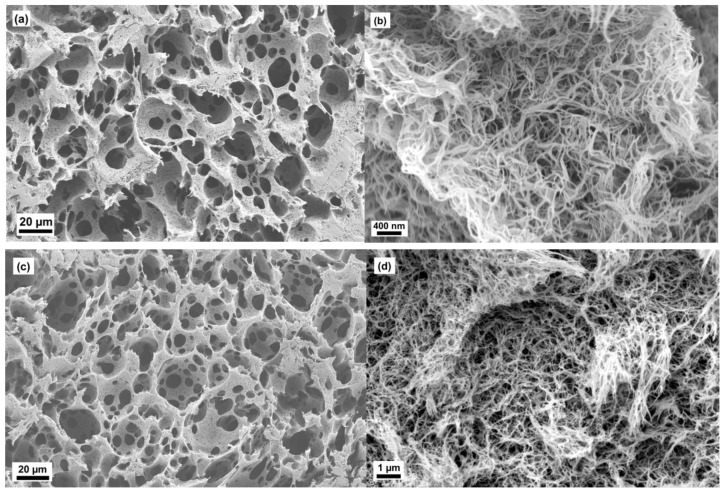
Scanning electron microscopic images of ACS-PN17 (**a,b**) and ACS-PS18 (**c,d**).

**Figure 5:**
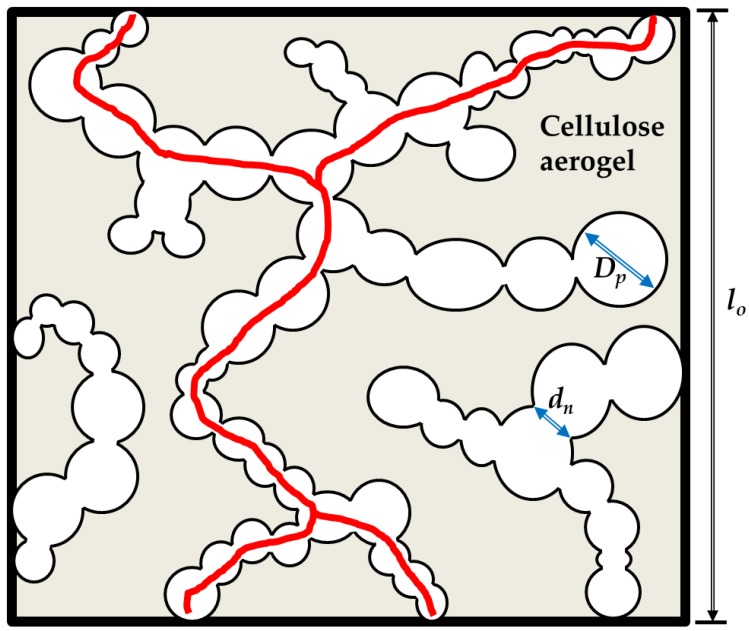
Schematic of the macropore space embedded in the cellulose aerogel network. The macropore stemming from oil drops are interconnected by necks with a neck size *d_n_*, being always smaller than the pore size *D_p_*. The red lines illustrate a possible gas flow path.

The microstructures of aerogels of scaffolds are shown in Figure 2, Figure 3 and Figure 4. The oil template produced secondary porous structure and they were interconnected due to the coalescence of the oil droplets during the synthesis part. These openings produced due to coalescence were named as neck parts (*d_n_*, see Figure 5). The neck diameter was always smaller than the secondary porous structure. The secondary porous structure varied in size and shape depending upon the physicochemical properties of the surfactants. The cell walls had the primary porous structure (that is usually found in classical cellulose aerogels).

In the case of ACS-PT13.5, a cellulose scaffold with a very wide distribution of macropore sizes, 50–600 µm was observed (Figure 2a,b). The thickness of the cell walls was varied which was in the range 750 nm–100 µm. Only few neck parts were noticed between the macropores and those were about 10–20 µm in size.

In the case of ACS-PO15, the macropore size distribution ranged from 100 to 400 µm (Figure 2c,d). It was confirmed in our earlier study [16] that all the secondary pores were interconnected by the neck opening, 50–100 µm. The size and shapes of pore channels varied and the secondary pore shapes seemed to be worm-like in structure. The cell wall thickness was in the range of between 35 and 175 µm.

In the case of ACS-PS15, it showed a microstructure with macropores of an average size of 75–150 µm, a cell wall thickness of 10–50 µm and a macroporosity of 60%. Figure 3a,b showed that the porous structures were inhomogeneously distributed with irregular shaped macropores. In comparison with ACS-PO15, ACS-PS15 showed smaller macropores 75–150 µm even though the surfactants, PO15 and PS15 had the same HLB value of 15. It can be reasoned out with the chemical structure of those surfactants. Both of them with the numbers of carbon (C_18_) and oxygen (O_21_) atoms in the molecular formula were same. The only exception was the number of hydrogen atoms, i.e., PO15 had two hydrogen atoms less than PS15. It resulted in a structural difference. PO15 had the cis-isomer of an oleyl-lipophilic backbone whereas PS15 had a linear stearyl-lipophilic backbone. During the synthesis of scaffolds, an oleyl-backbone with a cis-isomeric structure self-assembles in the interface between a cellulose solution and oil droplets and they are able to stabilize bigger oil droplets in comparison to a stearyl-backbone.

In the case of ACS-PC16, the microstructure was in the size range 50–110 µm and the neck diameters were 25–75 µm (Figure 3c,d). The total macroporosity was 54%. ACS-PC16 showed big microstructural difference when the images were compared with ACS-PS15 (Figure 3a–d). It can be reasoned out with the chemical environment of surfactants used. In comparison with the chemical structure of the surfactant PS15, PC16 had two carbon atoms less, that means it had a C_16_ linear carbon chain in its backbone. With this chemical difference, PC16 was more hydrophilic than PS15 though both had the same number of ethylene oxide chemical moieties.

In the case of ACS-PN17 and ACS-PS18, the microstructure was observed to be similar to the structures reported for high internal phase emulsion templating (HIPE) [21] (Figure 4). The macropores were close to spherical in shape and each macropore structure had many neck parts. The neck diameters were in the size range 4–20 µm in the case of ACS-PN17 whereas they were about 2–10 µm in the case of ACS-PS18. The cell wall thickness varied between a few hundred nanometers and a maximum of up to 3 µm. The total macroporosity from oil template method was about 71–72%.

It was interesting to compare and note down that PS18 and PS15 had the same lipophilic backbone and differed in the hydrophilic moiety, i.e., the number of ethylene oxide groups for PS15 was 20 whereas they were 100 for PS18. Scanning electron microscopic image data of the corresponding aerogels implied that the surfactants with a small difference in chemical moiety caould provide a huge microstructural difference in cellulose scaffolds production. It can also be concluded that the secondary porous structures can be reduced from 400 µm to 30 µm by choosing the surfactants with increasing HLB values from 15 to 18.

### 2.3. Gas Permeability Data Analysis

The macro–meso porous aerogels processed exhibited the following features with respect to gas (atmospheric air) flow through them:

- the pressure–time curves followed the theoretical model made for pure Darcy flow used to fit the curves and extract the permeability constant,
- the permeability was independent of the pressure difference and
- the permeability varied with the surfactant used.

These three observations show that Darcy’s law can be applied without taking into account special effects, like the Klinkenberg effect, slip flow at the cell walls or Knudsen effects [22,23,24]. A calculation of the permeability constant *K* for these aerogels seems to be straight forward and for instance the Carman-Kozeny model might be applied. This model reads:

*K* = (1/*τ*)(*φ_p_*^3^/180(1 − *φ_p_*)^2^)*D_p_*^2^ (1)

with *φ_p_* the pore volume fraction, *D_p_* the pore diameter and *τ* the tortuosity. A straight cylinder extending from one side of the sample to the other perpendicular would yield a tortuosity factor of 1, a very curved channel could yield much larger values of *τ*. In a simple picture *τ = l/l_o_*, in which *l_o_* is the thickness of the sample and *l* the real length of a pore. Unfortunately, *τ* is hard to measure and evaluate. A schematic of the pore space in the dual porous aerogels is shown in Figure 5, explaining also the tortuosity.

The permeability in this dual-pore material, in which we have macropores with a diameter in the range of a few 10 to a few 100 µm and mesoporous cell walls consisting of a nanofibril network, one might question if the Karman-Kozeny model applies. A simple extension would be to assume a parallel network of macropores with length *l* and cell walls enclosing them. Then the permeability can be expressed as:

*K_eff_ = K_c_φ_c_ + K_m_φ_m_* (2)

in which *K_c_* is the permeability of the pure cellulose aerogel making the cell walls and *K_m_* is the permeability of the macroporous material assuming that the walls do not allow any gas to permeate. In order to evaluate this equation, we need to have at least an estimate of the macropore volume fraction. In principle this could be calculated from the envelope densities of the cellulose aerogel and that of the macro–meso porous material, but in our samples this could not be done, since the cellulose aerogel in the cell walls had a different envelope density to the pure cellulose aerogels: the oil droplets squeezed the cell walls and increased their density.

In addition, as can be seen in the SEM pictures, the pore size of the macropores was different from the neck size of their interconnection, which was the area where two oil droplets met during processing and converted the dispersion of droplets into a more or less open porous network. The pore diameter to be inserted into the Carman-Kozeny equation was therefore not the macropore size, but the size of the necks *d_n_*. This is hard to measure, but we used again image analysis to extract from the SEM picture at least a good estimator of the neck size. The result is shown in Table 2 with an estimated error.

**Figure 6:**
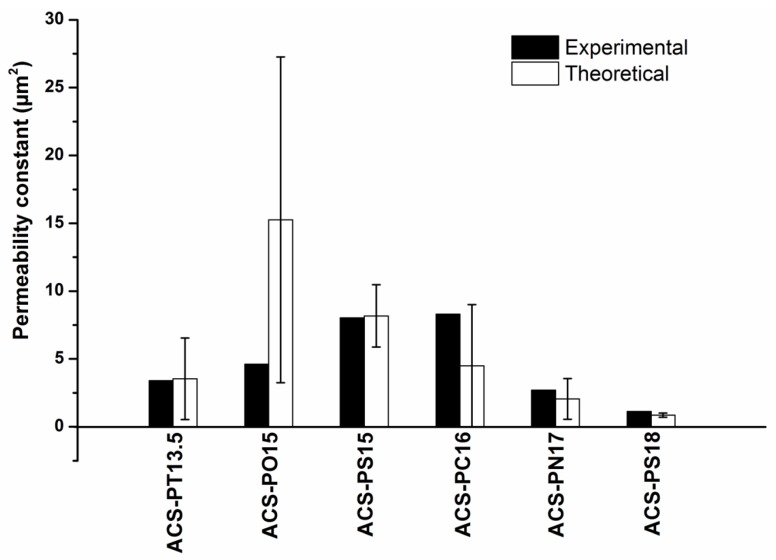
Experimental and calculated permeability according to Equation (1) with the data shown in Table 2. Error bars are shown, which reflect the uncertainty of the experimental data on pore volume fraction and neck diameter.

From our measurements on pure cellulose aerogels we know that the permeability is typically an order of magnitude smaller than the values measured here, see Table 3. We therefore neglected in a first approach to describe the measured permeability with a Carman-Kozeny law any effect the dual porosity might have induced. We calculated the permeability with Equation (1) and the values for the macropore volume fraction and the neck diameter and compared it to the experimental results. This yielded the values shown in Figure 6. Comparing the experimental and theoretical permeability data showed that for all samples the agreement was satisfactory in view of the uncertainties of the pore volume fraction and the neck diameter. For the sample ACS-PO15 there was a large discrepancy, which might have its origin in the tortuous network. The macropores were not connected via the necks in straight lines going through the sample in any of the samples, but the necks from macropore to macropore led the air flow in a tortuous way. It looks as if the tortuosity in the sample ACS-PO15 was much larger and thus led to a smaller value.

There is another aspect, which we have to discuss. The macropore fraction was in the range of 55 to 72% and thus much larger than expected. It points to the issue, if the oil droplets might have squeezed the cellulose gel to a higher density. In fact, a simple calculation can show this. The envelope density of macro–meso porous aerogels, *ρ^e^_mm_*, is given by the weighted superposition of the envelope density of the cellulose aerogel in the cell walls, *ρ^e^_cw_* and the density of air in the macropores *ρ_a_*

*ρ^e^_mm_ = φ_cw_ ρ^e^_cw_ + φ_cw_ ρ_a_* (3)

with *φ_cw,_ φ_m_* the volume fraction of cell walls and of the macropores. Using *φ_cw_ + φ_m_* = 1, we obtain for the envelope density of the cell walls (neglecting the density of air):

*φ^e^_cw_* ≅ *ρ^e^_mm_ /*(1 − *φ_m_*). (4)

**Figure 7:**
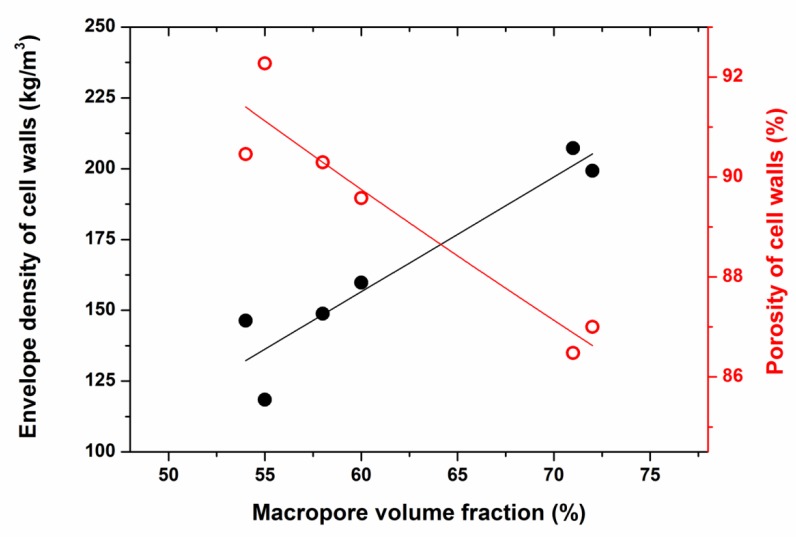
Envelope density of the cell walls (left axis) and their porosity (right axis) as it varied with the macropore volume fraction. The lines drawn in are guides for the eyes.

Figure 7 shows that the cell wall density increased with increasing macropore volume fraction. The more volume was occupied by the oil droplets the more the cellulose gel was compressed. This figure also shows that there still was a large porosity in the cell walls (87 to 91%).

If we apply the relation determined by Hoepfner [25] and Karadagli [26] between envelope density and the cellulose content in a solution to prepare a monolithic pure cellulose aerogel using the same salt-melt hydrate route applied here, one can calculate the amount of cellulose necessary to yield such high envelope densities. They would be in the range from 6 wt% to 10 wt%. Looking at the SEM pictures, such an apparent high solid content in the cell walls seems possible. We therefore suggest that incorporation of oil droplets in the cellulose solution led to a compaction of the cellulose fibrils in the cell walls between the droplets. Especially, in detail, this behaviour can be correlated with the surfactant’s properties. For instance, surfactants PN17 and PS18 with HLB values 17 and 18 stabilized the internal oil phase contents of >70% resulting in high internal phase separated emulsion. It led to the compact arrangement of cellulose chains with thick nanofibrillar diameter. That produced a finely distributed macropore structure with low specific surface area and large macropore volume fraction, high envelope cell wall densities and low porosity of cell walls (see Table 1 and Table 2, Figure 4 and Figure 7).

## 3. Materials and Methods

### 3.1. Synthesis of Cellulose Aerogels

The chemicals were purchased from Sigma-Aldrich and used as received. Microcrystalline cellulose (medium fibres, product number is C6288), calcium thiocyanate tetrahydrate (95%), glyceryl trioctanoate, polyoxyethylene tert-octylphenyl ether (Triton^TM^ X-100; PT13.5), polyoxyethylene (20) oleyl ether (Brij^®^ 98; PO15), polyoxyethylene (20) stearyl ether (Brij^®^ S 100; PS15), polyoxyethylene (20) cetyl ether (Brij^®^ 58; PC16), polyoxyethylene (40) nonylphenyl ether, branched (IGEPAL^®^ CO-890; PN17) and polyoxyethylene (100) stearyl ether (Brij^®^ S 100; PS18) were purchased from Sigma-Aldrich. The HLB values were obtained from the materials safety data sheet of the surfactants. In the abbreviations of surfactants, the HLB values were included. The aerogels and aerogel scaffolds were prepared by the methods reported by our earlier literature [16,17]. Aerogels of cellulose containing 2 wt%, 4 wt% and 6 wt% were abbreviated as AC-2, AC-4 and AC-6 respectively. Aerogels of cellulose scaffolds (ACS) prepared using six different surfactants, PT13.5, PO15, PS15, PC16, PN17 and PS18 were labelled as ACS-PT13.5, ACS-PO15, ACS-PS15, ACS-PC16, ACS-PN17 and ACS-PS18 respectively. In the gel preparation process deionized water was used. Cellulose (4 g) and calcium thiocyanate tetrahydrate (96 g) were mixed together with 80 mL of deionized water. The mixture was heated up to 117 °C for 60 min. Meanwhile, the glyceryl trioctanoate (oil) (96.5 g) and surfactant (0.5 g) were mixed together under stirring at 125 °C. This hot oil and surfactant mixture (100 g) was added to the cellulose solution once the dissolution of cellulose was confirmed. After 15 min stirring at 150 rpm, the creamy mixture was transferred to the moulds and cooled to room temperature. After 16 h ageing, the gel body was washed with acetone in order to remove oil and surfactant and then several times with ethanol. The traces of removal of calcium thiocyanate were confirmed as per the methods reported in literature [20]. The pure wet gels having ethanol were dried under super critical drying conditions. Supercritical drying was carried out in an autoclave using pure carbon dioxide, following the procedure reported by Hoepfner et al. [25].

### 3.2. Characterisation Methods

The products were characterized by envelope density measurement (Micromeritics–GeoPyc 1360), skeletal density (Micromeritics–Accupyc II 1340; Gas pycnometer–Helium), Brunauer-Emmett-Teller (BET) nitrogen adsorption–desorption isotherm analysis (Micromeritics–Tristar II 3020), scanning electron microscopy coupled with energy-dispersive X-ray spectroscopy (SEM: Merlin–Carl Zeiss Microscope; gold sputtered samples) and gas permeability measurements using an in-house designed facility, described in [27] and briefly sketched below. The microstructure was analysed on the SEM pictures using the ImageJ program by first thresholding the images to reveal the macropores and the cell walls. Then 10–15 equidistant lines were superimposed over the resulting binary image and the linear intercept in the macropores determined in the cell walls and the macroporosity was determined from the ratio of the line length in the pores in relation to the total line length on the image. In addition, the macroporosity was determined on the binary images by directly evaluating the number of pixels in the macropores and relating it to the total number of pixels in the image.

### 3.3. Experimental Set Up for Gas Permeability Measurement

**Figure 8:**
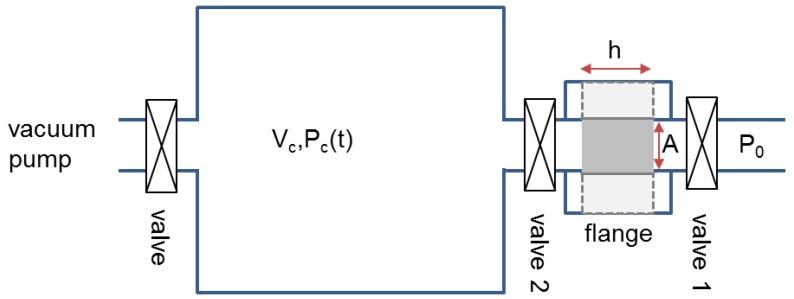
Experimental setup to measure the gas permeability. The samples had the thickness *h* and cross-sectional area *A*.

A key point in the preparation of a dual pore system aerogel, having macro- and mesopores, is to control with the macroporosity the permeability to gases and using the mesopores inside the cell walls as absorbers. We therefore developed a special set-up to measure the permeability and to show the effect of the mesopores on gas absorption. The set-up is sketched in Figure 8. Gas permeability is measured using different techniques. Torrent [28] describes a method especially developed for concrete, in which a chamber is placed tightly onto a concrete cover, evacuated and the pressure increase in this chamber measured. Other methods use a gas flow technique, meaning the gas current through a porous medium is varied and then evaluated with Darcy’s law to obtain the permeability [23,24,29,30,31,32].

In our facility the sample whose permeability shall be measured is mounted in a stainless steel ring and fixed therein with a special silicone gel such that no gas can pass the porous body aside but passes only through its pores. This steel ring is connected to a flange that itself is connected on both sides to valves (valve 1 and 2 in Figure 8). A cylindrical chamber of volume *V_c_* serves as a test volume. For the measurement the test volume is evacuated to a preset pressure *P_c_(0)* and the valve 1 in front of the sample is opened (while valve 2 always is open). Then the ambient pressure *P_0_*, being also measured, exists on one side of the sample and on the other side initially the lower pressure *P_c_*. The pressure difference drives a gas (atmospheric air) flow through the porous body. A vacuum gauge measures the pressure increase *P_c_(t)* in the chamber with time. The flow through the porous body is determined by its permeability *K*, which is fully determined by the microstructure of the porous body like pore volume fraction, pore size, pore morphology and others [23,24,29,31,32]. The permeability is directly attainable from the pressure change measurement. The relation between pressure change and permeability can be derived as follows. The gas flow through the body shall be described by Darcy’s law:

*u_D_* = − (*K*/*η*)(*dP*/*dx*). (5)

The flow velocity *u_D_* is proportional to the pressure gradient *dP*/*dx*, the dynamic viscosity *η* of the gas (typically of the order 10*^−^*^6^ Ns/m^2^) and the permeability *K* which has the unit (length)^2^. One can calculate the chamber pressure as a function of time in this set-up analytically (provided the flow is described by Darcy’s equation) and yields the following result [27]:

*P_c_(t)* = *P_0_* tan *h* ((A*KP_0_* / 2*ηhV_c_*) *t* + artan *h* (P_c_^0^ / P_c_)) = *P_0_* tan *h* ((*t* / *τ_c_*) + artan *h* (*P_c_^0^* / *P_c_*)), (6)

where *A* is the sample area and *h* its thickness. The characteristic time *τ_c_* can be expressed as:

*τ_c_* = 2*ηhV_c_* / A*KP_0_* (7)

The relation shows: the larger the ambient pressure, the shorter the time to raise the pressure in the chamber to its ambient value and the larger the chamber volume the larger the time. Thus, by increasing the volume *V_c_* or the sample thickness and reducing the ambient pressure *P_0_* (but keeping it constant) the measurement time can be increased. Measurement of the pressure increase as a function of time and fitting the resulting data points with the expression in Equation 6 allows the characteristic time *τ_c_* to be determined and thus the permeability as:

*K* = 2*ηhV_c_* / A*P_0_τ_c_* (8)

In our set-up we therefore have only a one parameter fit from the experimentally determined *P_c_(t)* curve, since only *τ_c_* is unknown; all other values are either preset or measured.

Using Equation (6), the theoretical and the experimental data values were compared for the samples ACS-PS18 at different set initial pressure values (Figure S1).

## 4. Conclusions

A variety of hierarchical porous structures of cellulose aerogels have been developed using six different surfactants. The structural properties of aerogels were highly influenced by the physico-chemical properties of the surfactants. Using higher the HLB values of surfactants, the finely organized macropores could be produced with compact arrangement of cellulose chains in the cell walls which resulted in high cell wall density, low specific surface area and large macropore volume fraction. Surfactants with different chemical, physical and structural properties play a significant role in determining the fabrication of macropore channels. For instance, PO15 with its cis-form produced bigger macropore channels (diameter of 100–400 µm) whereas PS15 with its straight chain produced smaller macropores (diameter of 75–150 µm). The difference in design of the macropore channel influenced the gas permeability values. The air flow through the macropore channels are depending upon the alignment of the macropore channels and the cell wall thickness composing mesopores which controls the turbidity, tortuosity, gas slippage, etc. In order to understand the gas flow properties in aerogels, more experiments are required preparing the well-ordered macropore channels and comparing the properties with these materials prepared in the present studies. The Carman-Kozeny model fits well for the analysis of dual porous aerogel materials. The structured dual porous materials can reduce the air flow resistance under certain pressure. In comparison with the classical cellulose aerogels, AC-2 had a similar envelope density value; the ACS materials with secondary porous structures improved the gas permeability: 24 times in the case of ACS-PS18 and 178 times in the case of ACS-PC16. In the future design of advanced filter/membranes production, biological or catalytic supporting materials, the reported emulsion method can be adapted and a wide range of hierarchical structural materials can be prepared.

## Figures and Tables

**Table 1:** Summary of the physical properties of cellulose materials. The skeletal density of cellulose II was taken from our previous report [16] which was about 1530 kg/m^3^.

Sample	Envelope Density, *ρ_e_* (kg/m^3^)	Total Porosity (%)	BET Specific Surface Area (m^2^/g)
AC-2	63 ± 0.15	95.9	287
AC-4	108.8 ± 12.4	92.9	314
AC-6	137.9 ± 1	91.0	282
ACS-PT13.5	62.5 ± 2.5	95.9	304
ACS-PO15	53.3 ± 0.9	96.4	301
ACS-PS15	63.9 ± 0.2	95.8	327
ACS-PC16	67.3 ± 1	95.6	296
ACS-PN17	60.1 ± 0.9	96.1	262
ACS-PS18	55.8 ± 2.3	96.3	245

**Table 2:** Summary of microstructural parameters of the various dual pore cellulose materials.

Sample	Volume Fraction of Macropores	Secondary Pore Diameter, µm	Neck Diameter, µm	Cell Wall Thickness, µm
ACS-PT13.5	58 ± 4	50–600	10–20	0.75–100
ACS-PO15	55 ± 4	100–400	50–100	35–175
ACS-PS15	60 ± 4	75–150	20–75	10–50
ACS-PC16	54 ± 4	50–110	10–40	10–20
ACS-PN17	71 ± 4	30–40	4–20	1–3
ACS-PS18	72 ± 4	20–30	2–10	0.25–2

**Table 3:** Permeability data of the different samples. Average for three initial pressure differences (300,500,700 mbar) with their standard deviation.

Sample	Permeability Constant, *K*, µm^2^
AC-2	0.0465 ^a^
AC-4	0.005 ^a^
AC-6	0.0036 ^a^
ACS-PT13.5	3.40 ^b^
ACS-PO15	4.61 ^b^
ACS-PS15	8.02 ^b^
ACS-PC16	8.30 ^b^
ACS-PN17	2.70 ^b^
ACS-PS18	1.13 ^b^

^a^ These values were obtained at initial pressure difference of 500 mbar. ^b^ These were average values obtained from the initial pressure difference of 300, 500 and 700 mbar (see Figure S2).

## Supplementary Materials

The change in chamber pressure from the set initial pressure indicates that the gas molecules pass through the aerogel sample with respect to time and comes to the equilibrium. Figure S1 shows the chamber pressure as a function of time for a dual pore system aerogel (ACS-PS18) which was prepared using PS18 surfactant. The theoretical calculation fits almost to the experimental data employing Equation (6) (see the open circles in Figure S1). **Figure S1.** Chamber Pressure as a function of time for a dual pore system aerogel (ACS-PS18) treated with PS18 surfactant. Four curves are shown which differ by the initial pressure in the chamber and thus the pressure difference applied to the sample. For the curve with the biggest pressure difference the data (open circles) were fit with the prediction of Equation (6). There was an almost perfect agreement between theory and experimental data showing that the theoretical model behind the evaluation of the permeability constant was valid. The gas permeability values of aerogels of cellulose scaffolds (ACS) at different chamber pressure values are shown in Figure S2. The average values of these are mentioned in Table 3. **Figure S2.** Gas permeability data of aerogels of cellulose scaffolds (ACS).
