# Supplementary material for: Gas Permeability of Cellulose Aerogels with a Designed Dual Pore Space System
Source: Molecules. 2019 Jul 24;24(15):2688. doi: 10.3390/molecules24152688 (PMC6696266; doi:10.3390/molecules24152688)
Supplement: Supplementary file 1 [file molecules-24-02688-s001.pdf]

molecules

MDPI

Type of the Paper (Article)

Gas permeability of cellulose aerogels with a designed dual pore space system

Kathirvel Ganesan [1], Adam Barowski [1] and Lorenz Ratke [1,*]

[1] Institue of Materials Research, German Aerospace Center (DLR), Linder Höhe, 51170 Köln, Germany; Lorenz.Ratke@gmx.de

* Correspondence: Lorenz.Ratke@gmx.de; (L.R.)

Received: 2 July 2019; Accepted: 22 July 2019; Published: 24 July 2019

Supplementary Materials:

The change in chamber pressure from the set initial pressure indicates that the gas molecules pass through the aerogel sample with respect to time and comes to the equilibrium. Figure S1 shows the chamber pressure as a function of time for a dual pore system aerogel (ACS-PS18) which was prepared using PS18 surfactant. The theoretical calculation fits almost to the experimental data employing equation (6) (see the open circles in Figure S1).

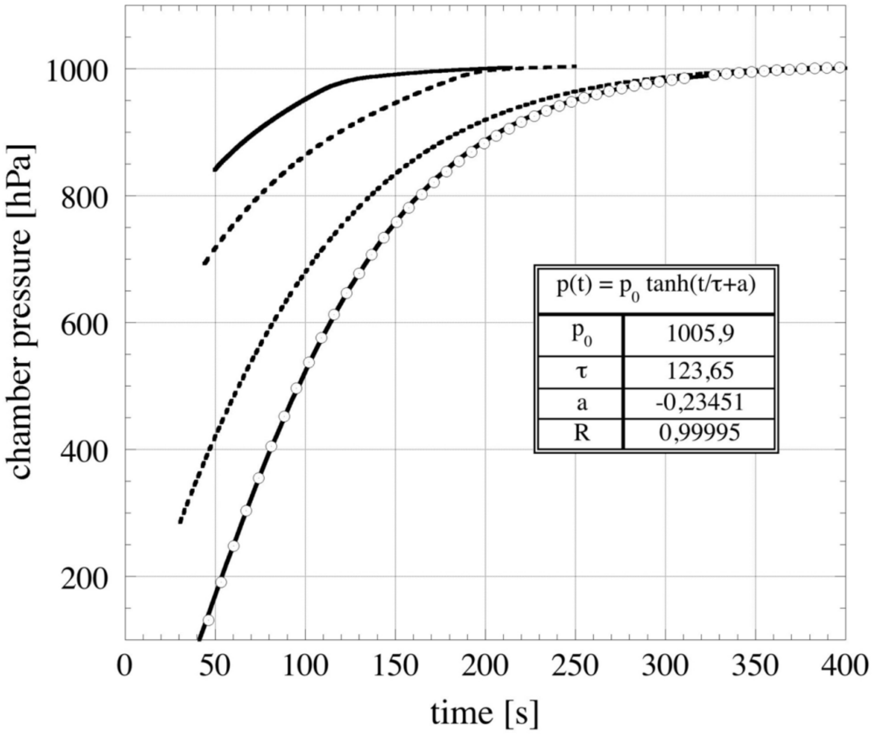

Figure S1. Chamber Pressure as a function of time for a dual pore system aerogel (ACS-PS18) treated with PS18 surfactant. Four curves are shown which differ by the initial pressure in the chamber and thus the pressure difference applied to the sample. For the curve with the biggest pressure difference the data (open circles) are fit with the prediction of equation 6. There is an almost perfect agreement between theory and experimental data showing that the theoretical model behind the evaluation of the permeability constant is valid.

The gas permeability values of aerogels of cellulose scaffolds (ACS) at different chamber pressure values are shown in Figure S2. The average values of these are mentioned in Table 3.

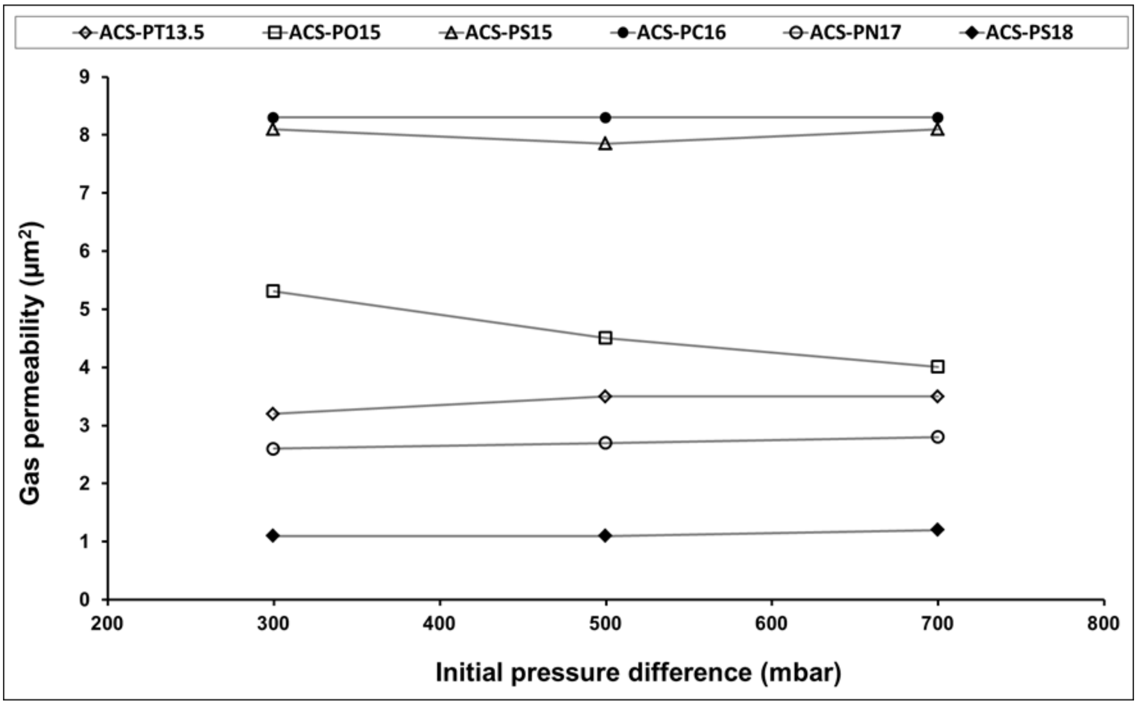

Figure S2. Gas permeability data of aerogels of cellulose scaffolds (ACS).
